# Inflammatory Gene Expression in Whole Peripheral Blood at Early Stages of Sporadic Amyotrophic Lateral Sclerosis

**DOI:** 10.3389/fneur.2017.00546

**Published:** 2017-10-13

**Authors:** Pol Andrés-Benito, Jesús Moreno, Raúl Domínguez, Ester Aso, Mónica Povedano, Isidro Ferrer

**Affiliations:** ^1^Neuropathology, Pathologic Anatomy Service, Bellvitge University Hospital, IDIBELL, Hospitalet de Llobregat, Spain; ^2^Biomedical Network Research Center on Neurodegenerative Diseases (CIBERNED), Institute Carlos III, Hospitalet de Llobregat, Spain; ^3^Functional Unit of Amyotrophic Lateral Sclerosis (UFELA), Service of Neurology, Bellvitge University Hospital, Hospitalet de Llobregat, Spain; ^4^Department of Pathology and Experimental Therapeutics, University of Barcelona, Hospitalet de Llobregat, Spain; ^5^Institute of Neurosciences, University of Barcelona, Hospitalet de Llobregat, Spain

**Keywords:** amyotrophic lateral sclerosis, blood, cytokines, extracellular matrix, leukocyte extravasation

## Abstract

**Objective:**

Characterization of altered expression of selected transcripts linked to inflammation in the peripheral blood of sporadic amyotrophic lateral sclerosis (sALS) patients at early stage of disease to increase knowledge about peripheral inflammatory response in sALS.

**Methods:**

RNA expression levels of 45 genes were assessed by RT-qPCR in 22 sALS cases in parallel with 13 age-matched controls. Clinical and serum parameters were assessed at the same time.

**Results:**

Upregulation of genes coding for factors involved in leukocyte extravasation (*ITGB2, INPP5D, SELL*, and *ICAM1*) and extracellular matrix remodeling (*MMP9* and *TIMP2*), as well as downregulation of certain chemokines (*CCL5* and *CXC5R*), anti-inflammatory cytokines (*IL10, TGFB2*, and *IL10RA*), pro-inflammatory cytokines (*IL-6*), and T-cell regulators (*CD2* and *TRBC1*) was found in sALS cases independently of gender, clinical symptoms at onset (spinal, respiratory, or bulbar), progression, peripheral leukocyte number, and integrity of RNA. *MMP9* levels positively correlated with age, whereas *CCR5, CCL5*, and *TRBC1* negatively correlated with age in sALS but not in controls. Relatively higher *TNFA* expression levels correlate with higher creatinine kinase protein levels in plasma.

**Conclusion:**

Present findings show early inflammatory responses characterized by upregulation of factors enabling extravasation of leukocytes and extracellular matrix remodeling in blood in sALS cases, in addition to increased *TNFA* levels paralleling skeletal muscle damage.

## Introduction

Increase in the number of astrocytes and microglia, and activation of inflammatory responses are major pathological marks in the anterior horn of the spinal cord in amyotrophic lateral sclerosis (ALS). Chronic inflammation plays the principal role in motor neuron demise and parallels the severity of motor neuron damage. A plethora of receptors, modulatory factors, chemokines, and anti- and pro-inflammatory cytokines are involved in this process at advanced stages of the disease (1–7). Inflammatory responses in the central nervous system are accompanied by modifications in blood and serum which may indicate a systemic inflammatory response in ALS (8–11). Peripheral nerves, autonomic nervous system, and muscle are involved in ALS, and they are putative targets of inflammatory reactions (12–18). Recent studies have also shown modifications in the intestinal microbiota in ALS (19, 20), thus categorizing ALS as a disease with multisystem involvement.

The majority of studies of blood and serum in ALS are at middle or advanced stages of the disease with or without treatment (10, 17, 21–26), but information about early stages at the time when the patient first asks for medical counseling and the disease is then diagnosed is limited (27). The purpose of the present study was to increase knowledge about expression of transcripts linked to inflammation in whole blood samples of sporadic ALS (sALS) patients at initial clinical stages of the disease. The selection of genes was conducted including representative pro- and anti-inflammatory cytokines, chemokines, cytokine modulators, extracellular matrix remodeling-related factors, molecules involved in extravasation mechanisms, oxidative stress markers, and T-cell regulators. The expression of these molecules was assessed considering the variables RNA integrity, gender, clinical symptoms at onset (spinal, respiratory, or bulbar), disease progression, peripheral leukocyte number, and creatinine kinase protein levels in plasma.

## Materials and Methods

### Sample Description

Whole peripheral blood samples for mRNA expression and biochemical studies were obtained within the two first months after the diagnosis. Samples were obtained from 22 sALS patients (mean age at plasma sampling 62.5 years; 16 men and 6 women) and 13 healthy age-matched controls (mean age at plasma sampling 65 years; 11 men and 4 women). sALS patients were selected on the basis of early stage at the diagnosis with homogenous parameters of gender, age, and treatment, whereas controls were recruited on the basis of homogenous parameters of gender and age. Patients were evaluated clinically according to the main signs at onset (spinal, bulbar, and respiratory) and categorized according to disease progression as fast, expected, and slow progression depending on the survival or the clinical evolution in those still alive. Fast progression was considered in patients who survived less than 3 years; expected progression was considered between 3 and 5 years, and slow for those still alive after 5 years. The ALS Functional Rating Scale Revised (ALS-FRS-R, version May 2015) was currently used in every case. No ALS cases or controls suffered from infection or inflammatory disorder at the time of sampling. None of them complained of systemic disease and none received any treatment related to ALS. No familial forms of ALS for *C9ORF72, SOD1, TARDBP*, and *FUS* mutations were detected when DNA of each patient was sequenced. Blood samples from sALS cases and age-matched controls were obtained following signed informed consent and approval by Clinical Research Ethics Committee (CEIC) of the Bellvitge University Hospital. A summary of cases is shown in Table 1.

**Table 1:** Summary of cases analyzed in the present study.

Case	Age at plasma sampling	Gender	Diagnosis	Initial symptoms	RIN value
1	60	M	Control	–	9.1
2	68	M	Control	–	9.2
3	66	F	Control	–	9.0
4	N/A	M	Control	–	8.9
5	74	M	Control	–	8.0
6	N/A	F	Control	–	8.3
7	76	F	Control	–	7.8
8	67	M	Control	–	6.1
9	72	F	Control	–	6.0
10	44	F	Control	–	6.0
11	66	F	Control	–	6.1
12	62	F	Control	–	6.5
13	63	F	Control	–	6.0
14	60	M	ALS	Spinal	7.4
15	63	M	ALS	Spinal	8.7
16	66	F	ALS	Bulbar	8.9
17	53	F	ALS	Bulbar	7.3
18	73	M	ALS	Bulbar	8.6
19	65	M	ALS	Spinal	8.9
20	43	M	ALS	Bulbar	8.6
21	57	F	ALS	Bulbar	7.4
22	65	M	ALS	Bulbar	7.1
23	67	M	ALS	Bulbar	7.4
24	73	M	ALS	Spinal	6.1
25	73	F	ALS	Spinal	6.0
26	59	F	ALS	Spinal	8.7
27	65	M	ALS	Respiratory	7.1
28	42	M	ALS	Bulbar	9.2
29	75	M	ALS	Respiratory	8.1
30	75	M	ALS	Bulbar	7.9
31	29	M	ALS	Spinal	8.3
32	77	M	ALS	Spinal	7.4
33	55	M	ALS	Spinal	8.5
34	69	M	ALS	Spinal	8.6
35	71	F	ALS	Spinal	8.7

*ALS, amyotrophic lateral sclerosis; M, male; F, female; RIN, RNA integrity number*.

### Blood Collection

In addition to current blood samples for hemogram and biochemical parameters, whole blood samples were collected using PAXgene Blood RNA Tube (PAXgene Blood RNA Tube, PreAnalytiX, Qiagen^®^ GmbH, Hilden, GE) collecting system. Two PAXgene Blood RNA tubes were obtained per case. Samples were collected at the first visit once the clinical diagnosis was established. Tubes were kept for 2 h at room temperature to ensure lysis of blood cells and then stored at −20°C for 24 h. Thereafter, tubes were transferred to −80°C for at least 7 days prior to processing.

### White Blood Cells (WBC) Counting

Blood was collected in EDTA 3 mL tubes and analyzed using flow-cytometry equipment. Technicon H-1, H-2, and H-3 apparatuses are discrete analyzers that perform complete blood and platelet counts, and leukocyte differential count. The instrument has a tungsten halogen light source and cytometer for leukocyte peroxidase analysis, with the addition of a helium-neon red laser for RBC/platelet and basophil determinations. Red blood cells are lysed, and fixed leukocytes flow in a stream sheath—a layer of inert liquid of the same refractive index. The stream sheath serves to narrow the sample stream, which prevents clogging and keeps the flow cell clean. Within the cell flow, cells are classified one by one on the basis of size (determined by a dark-field light scatter detector) and cytochemical peroxidase reaction. Measurement of the peroxidase activity is sufficient for most of the WBC differential classification. Lymphocytes are identified as small, unstained cells. Large atypical lymphocytes, plasma cells, and some blasts are characterized as “large unstained cells” (LUCs). Eosinophils exhibit the strongest peroxidase activity and appear smaller than neutrophils because they absorb some of their own scatter signal. Neutrophils are large and have moderate peroxidase activity. Monocytes have somewhat weaker peroxidase staining and are, therefore, in the area to the left of the neutrophils and to the right of the LUCs. The instrument’s computer automatically performs cluster analysis of the WBC subpopulations. The Technicon systems provide both relative (per cent) and absolute (×10^9^ cells/L) cell counts for neutrophils, eosinophils, basophils, monocytes, and LUCs.

### Quantitative Determination of Creatine Kinase (CK) in Blood Samples

Kinetic determination of CK was based upon IFCC (International Federation of Clinical Chemistry and Laboratory Medicine) and DGKC (Deutsche Gesellschaft für Klinische Chemie). The principle of the method is based on the ability of CK to catalyze the conversion of creatine phosphate and ADP to creatine and ATP. ATP and glucose are converted to ADP and glucose-6-phosphate by hexokinase. Glucose-6-phosphate dehydrogenase oxidizes glucose-6-phosphate to 6-phosphogluconate, reducing NADP to NADPH. The rate of conversion of NADP/NADPH, monitored at 340 nm, is proportional to CK activity. *N*-acetyl cysteine (NAC) is added as an activator of CK (28, 29).

### RNA Extraction and RT-qPCR

PAXgene Blood RNA tubes were incubated overnight at 4°C in a shaker-plate to equilibrate the temperature and increase yields and then at room temperature for 2 h before starting the procedure. RNA from frozen whole blood samples was extracted following the instructions of the supplier (PAXgene Blood RNA kit, PreAnalytiX, Qiagen^®^ GmbH, Hilden, GE). RNA integrity number (RIN) and 28S/18S ratios were determined with the Agilent Bioanalyzer (Agilent Technologies Inc., Santa Clara, CA, USA) to assess RNA quality. RNA concentration was evaluated using a NanoDrop™ Spectrophotometer (Thermo Fisher Scientific, Carlsbad, CA, USA). RIN values are shown in Table 1. Complementary DNA (cDNA) was prepared using the High-Capacity cDNA Reverse Transcription kit (Applied Biosystems, Foster City, CA, USA) following the protocol provided by the supplier. Parallel reactions for each RNA sample were run in the absence of MultiScribe Reverse Transcriptase to assess lack of genomic DNA contamination. TaqMan RT-qPCR assays were performed in duplicate for each gene on cDNA samples in 384-well optical plates using an ABI Prism 7900 Sequence Detection system (Applied Biosystems, Life Technologies, Waltham, MA, USA). For each 10 µL TaqMan reaction, 4.5 µL cDNA was mixed with 0.5 µL 20× TaqMan Gene Expression Assays and 5 µL of 2× TaqMan Universal PCR Master Mix (Applied Biosystems). The identification numbers and names of TaqMan probes are shown in Table 2. Probes were selected on the basis of our previous observations of inflammatory changes in the spinal cord and frontal cortex in sALS (7) together with additional markers linked to extravasation mechanisms and extracellular matrix remodeling. Mean values of two house-keeping genes, glucuronidase beta (*GUS-*β) (30) and glyceraldehyde 3-phosphate dehydrogenase (*GAPDH*) (31), were used as internal controls for normalization. The reactions were carried out using the following parameters: 50°C for 2 min, 95°C for 10 min, and 40 cycles at 95°C for 15 s, and at 60°C for 1 min. Finally, all TaqMan PCR data were captured using the Sequence Detection Software (SDS version 2.2.2, Applied Biosystems). Samples were analyzed with the double-delta cycle threshold (ΔΔCT) method.

**Table 2:** Genes, gene symbols, and references in the present series.

Gene	Gene symbol	Reference
Catalase	*CAT*	Hs00156308_m1
Cathepsin C	*CTSC*	Hs00175188_m1
Cathepsin S	*CTSS*	Hs00356423_m1
CD4 molecule/T-cell surface glycoprotein CD4	*CD4*	Hs01058407_m1
CD44 molecule	*CD44*	Hs01075861_m1
CD8a molecule/T-cell surface glycoprotein CD8a Chain	*CD8A*	Hs00233520_m1
Chemokine (C–C motif) ligand 5	*CCL5*	Hs00982282_m1
Chemokine (C–C motif) receptor 5	*CCR5*	Hs00152917_m1
Chemokine (C–X–C motif) receptor 5	*CXCR5*	Hs00173527_m1
Colony stimulating factor 1 receptor	*CSF1R*	Hs00911250_m1
Colony stimulating factor 3 receptor (granulocyte)	*CSF3R*	Hs00167918_m1
C-type lectin domain family 7 member A	*CLEC7A*	Hs01124746_m1
C–X–C motif chemokine ligand 8	*CXC8*	Hs00174103_m1
Glyceraldehyde-3-phosphate dehydrogenase	*GAPDH*	Hs02786624_g1
Inositol polyphosphate-5-phosphatase D	*INPP5D*	Hs00183290_m1
Integrin subunit beta 2	*ITGB2*	Hs00164957_m1
Integrin subunit beta 4	*ITGB4*	Hs00173995_m1
Intercellular adhesion molecule 1	*ICAM-1*	Hs00164932_m1
Intercellular adhesion molecule 5	*ICAM-5*	Hs00170285_m1
Interferon, gamma	*INFG*	Hs00989291_m1
Interleukin 1 beta	*IL1B*	Hs01555410_m1
Interleukin 10	*IL10*	Hs00961622_m1
Interleukin 10 receptor subunit alpha	*IL10RA*	Hs00155485_m1
Interleukin 10 receptor subunit beta	*IL10RB*	Hs00988697_m1
Interleukin 6	*IL6*	Hs00985639_m1
Interleukin 6 signal transducer	*IL6ST*	Hs00174360_m1
LFA-3 receptor	*CD2*	Hs00233515_m1
Lymphocyte function-associated antigen 1	*LFA-1*	Hs00158218_m1
Macrophage inflammatory protein 1-alpha	*CCL3*	Hs00234142_m1
Membrane-associated ring finger (C3HC4) 9	*MARCH9*	Hs04189729_m1
Monocyte chemotactic and activating factor	*CCL2*	Hs00234140_m1
Metallopeptidase-9	*MMP9*	Hs00234579_m1
Osteopontin	*SPP1*	Hs00959010_m1
Programmed cell death 1 ligand 2	*PD1L2*	Hs01057777_m1
Selectin L	*SELL*	Hs00174151_m1
Superoxide dismutase 1, soluble	*SOD1*	Hs00533490_m1
Superoxide dismutase 2, mitochondrial	*SOD2*	Hs00167309_m1
T cell receptor beta constant 1	*TRBC1*	Hs01588269_g1
TIMP metallopeptidase inhibitor 1	*TIMP-1*	Hs00171558_m1
TIMP metallopeptidase inhibitor 2	*TIMP-2*	Hs01091317_m1
Toll-like receptor 2	*TLR2*	Hs00610101_m1
Toll-like receptor 3	*TLR3*	Hs01551078_m1
Toll-like receptor 4	*TLR4*	Hs01060206_m1
Toll-like receptor 7	*TLR7*	Hs00152971_m1
Tumor growth factor B1	*TGFB1*	Hs00998133_m1
Tumor growth factor B2	*TGFB2*	Hs00234244_m1
Tumor necrosis factor receptor superfamily member 1A	*TNFRSF1*	Hs01042313_m1
Tumor necrosis factor-alpha	*TNFA*	Hs01113624_g1
Vascular endothelial growth factor A	*VEGFA*	Hs00900055_m1
β-Glucuronidase	*GUS-*β	Hs00939627_m1

### Statistical Analysis

The normality of distribution of fold change values was analyzed with the Kolmogorov–Smirnov test. The non-parametric Mann–Whitney test was performed to compare each group when values did not follow a normal distribution, whereas the unpaired *t*-test was used for normal variables. Statistical analysis and graphic design were performed with GraphPad Prism version 5.01 (La Jolla, CA, USA). Results were analyzed with Student’s *t*-test. Outliers were detected using the GraphPad software QuickCalcs (*p* < 0.05). The data were expressed as mean ± SEM and significance levels were set at *p < 0.05 and **p < 0.01 and ***p < 0.001, and tendencies at ^#^<0.1. Pearson’s correlation coefficient was used to assess a possible linear association between two continuous quantitative variables.

## Results

### General Clinical and Hematological Findings

Amyotrophic lateral sclerosis progression was heterogeneous in the present series. Hemogram was not altered in sALS patients with the exception of a few cases in whom slight increase of neutrophils and low levels of lymphocytes was observed. CK levels were out of range in some patients and moderately increased in a few sALS cases. Clinical, hematological, and biochemical data are summarized in Table 3.

**Table 3:** Biochemical alterations in blood samples of sporadic amyotrophic lateral sclerosis (sALS) cases.

sALS case	Clinical progression	Creatinine kinase (CK) (μkat/L)	Leukocyte populations (×10E9cells/L)				
			Neutrophil (1.5–5.7)	Lymphocyte (1.3–3.4)	Monocyte (0.31–0.92)	Eosinophil (0.03–0.39)	Basophil (0.01–0.09)
14	Expected	13.9^a^ (≤4.50)	3.7	1.4	0.48	0.02^b^	0.02
15	Expected	5.5^a^ (≤4.50)	3.4	2.1	0.46	0.21	0.04
16	Expected	3.5^a^ (≤2.30)	6.9^a^	0.8^b^	0.37	0.04	0.04
17	Fast	5.0^a^ (≤2.30)	4.2	1.0^b^	0.34	0.1	0.05
18	Fast	3.5 (≤4.50)	N/A	N/A	N/A	N/A	N/A
19	Slow	0.8 (≤4.50)	4.3	1.2	0.44	0.16	0.04
20	Fast	4.6^a^ (≤4.50)	N/A	N/A	N/A	N/A	N/A
21	Expected	5.9^a^ (≤4.50)	3.3	1.0^b^	0.30^b^	0.15	0.04
22	Expected	2.2 (≤4.50)	3.9	2.5	0.56	0.34	0.01
23	Expected	2.6 (≤4.50)	7.8^a^	1.0^b^	0.6	0.01^b^	0.03
24	Expected	2.8 (≤4.50)	6.6^a^	1.8	0.6	0.15	0.06
25	Fast	0.7 (≤4.50)	5.6	1.4	0.87	0.39	0.09
26	Fast	N/A	3.6	1.6	0.49	0.11	0.02
27	Expected	8.3^a^ (≤4.50)	7.0^a^	1.7	0.53	0.11	0.07
28	Fast	1.8 (≤4.50)	4.2	3.1	0.76	0.28	0.04
29	Fast	N/A	6.0^a^	0.9^b^	0.74	0.08	0.03
30	Fast	3.0 (≤4.50)	4.3	1.4	0.77	0.04	0.04
31	Fast	2.1 (≤4.50)	4.2	2.1	0.63	0.19	0.08
32	Fast	N/A	N/A	N/A	N/A	N/A	N/A
33	Slow	2.0 (≤4.50)	3.8	2.5	0.44	0.55	0.06
34	Slow	3.5 (≤4.50)	3.5	1.8	0.38	0.19	0.06
35	Fast	11.6^a^ (≤4.50)	N/A	N/A	N/A	N/A	N/A

*N/A, data not available; μkat/L, microkatals/liter*.

*Normal CK levels in brackets (these are variable depending on the method used; CK values in every ALS case are evaluated according to the method used)*.

*^a^Above normal range*.

*^b^Below normal range*.

### Gene Expression Levels

#### Anti-inflammatory Cytokines

**Figure 1:**
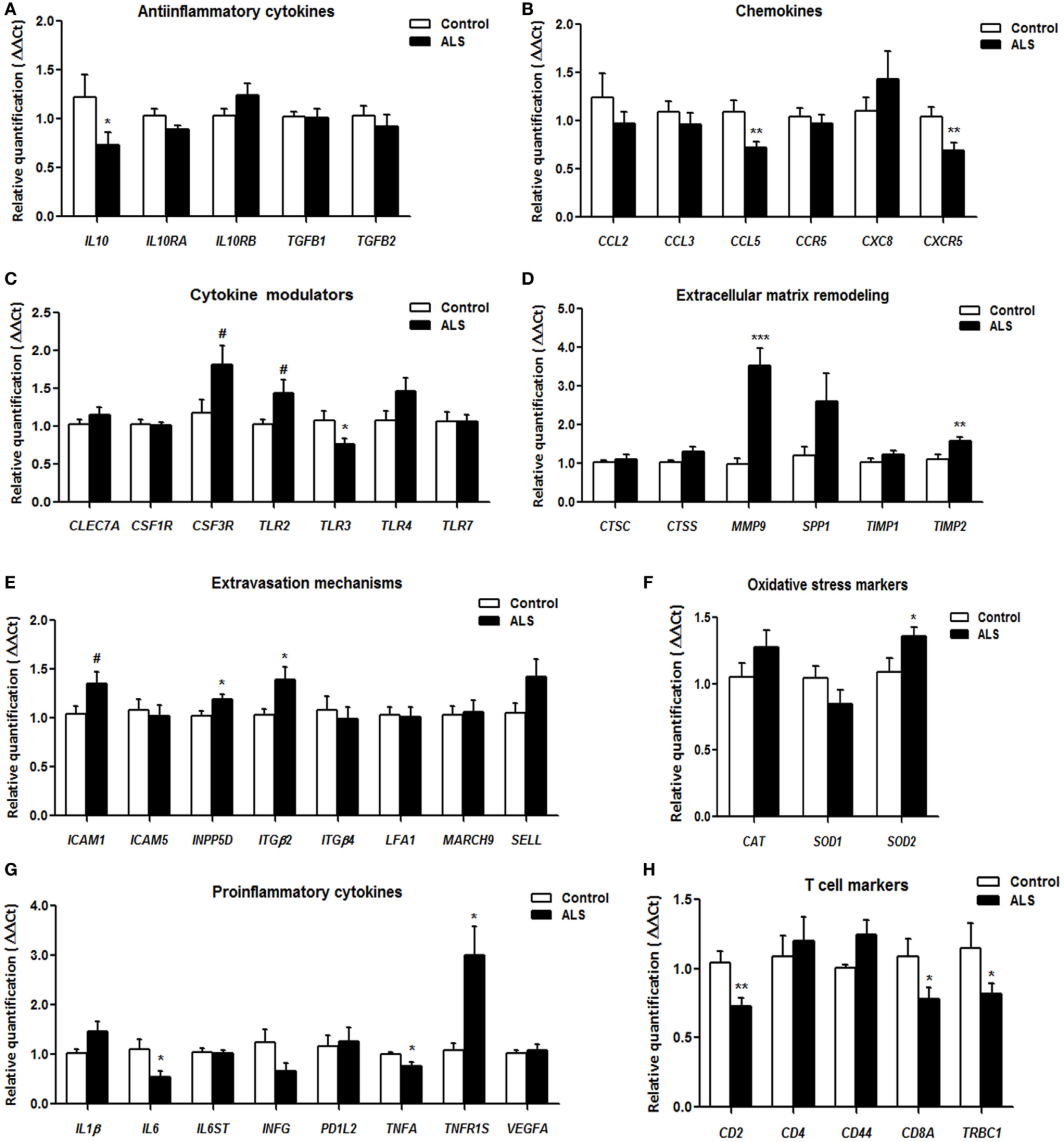
Gene expression of anti-inflammatory cytokines **(A)**, chemokines **(B)**, cytokine modulators **(C)**, extracellular matrix remodeling-related factors **(D)**, molecules involved in extravasation mechanisms **(E)**, oxidative stress markers **(F)**, pro-inflammatory cytokines **(G)**, and T-cell markers **(H)**, as revealed by RT-qPCR, in blood from control and sporadic amyotrophic lateral sclerosis (sALS) cases. All data were expressed as the mean ± SEM. Statistical comparisons were performed using unpaired *t*-test; significance level was set at *p < 0.05, **p < 0.01 and ***p < 0.001, and tendencies at ^#^<0.1. A total of 13 healthy samples and 22 sALS samples were included in RT-qPCR analysis.

*IL10*, coding for interleukin 10, and *TGFB2*, coding for transforming growth factor beta 1, mRNA levels were significantly reduced in sALS, whereas *IL10RA* which codes for interleukin 10 receptor subunit alpha showed a tendency to decrease. Expression levels of *IL10RB* and *TGFB1* encoding interleukin 10 receptor subunit beta and transforming growth factor beta 1, respectively, were not modified (Figure 1A).

#### Chemokines

Expression levels of *CCL5* and *CXC5R*, which code for C-C motif chemokine ligand 5 and C-X-C motif chemokine receptor 5, respectively, were significantly decreased; *CCR5* coding for C-C motif chemokine receptor 5 showed a tendency to decrease. No modifications were seen for C-C motif chemokine ligand 2 (*CCL2)* and 3 (*CCL3*), and C-X-C motif chemokine 8 (*CXC8*) (Figure 1B).

#### Cytokine Modulators

Toll like receptors *TLR2* and *TLR4* mRNA expression showed a tendency to increase, whereas *TLR3* mRNA expression was significantly decreased in sALS. *TLR7* and other genes involved in cytokine modulation such as C-type lectin domain family 7 member A (*CLEC7A*), colony stimulating factor 1 receptor (*CSF1R*), and colony stimulating factor 3 receptor (*CSF3R*) were not altered (Figure 1C).

#### Extracellular Matrix Remodeling

*MMP9*, coding for matrix metallopeptidase 9, and *TIMP2*, coding for its inhibitor protein, TIMP metallopeptidase inhibitor 2, were significantly increased in sALS. The expression levels of *CTSC, CTSS, TIMP1*, and *SPP1*, coding for cathepsin C, cathepsin S, TIMP metallopeptidase inhibitor 1, and osteopontin, respectively, were similar in sALS and controls (Figure 1D).

#### Extravasation Mechanisms

*ITGB2*, coding for integrin subunit beta 2, and *INPP5D*, coding for inositol polyphosphate-5-phosphatase D, were upregulated in sALS. Tendency to increase was found for *SELL* and *ICAM1*, coding for selectin-L and intercellular adhesion molecule 1, respectively. No changes were detected in the expression of *ICAM5, ITGB4, LFA1*, and *MARCH9* encoding, respectively, intercellular adhesion molecule 5, integrin subunit beta 4, lymphocyte function-associated antigen 1, and membrane associated ring-CH-type finger 9 (Figure 1E).

#### Oxidative Stress Markers

Expression of catalase (*CAT*) and superoxide dismutase 1 (*SOD1*) genes was not modified. Superoxide dismutase 2 (*SOD2*) showed a tendency to increase in sALS (Figure 1F).

#### Pro-inflammatory Cytokines

*IL6*, coding for interleukin-6, was significantly downregulated in sALS cases. TNF-α coding gene *TNFA* showed a tendency to decrease. In contrast, *TNFR1S*, the gene coding for its receptor, was significantly increased. No alterations were found in the remaining assessed genes *IL1B, IL6ST, INFG, PD1L2*, and *VEGFA*, coding for interleukin 1B, interleukin 6 signal transducer, interferon gamma, programmed cell death 1 ligand 2, and vascular endothelial growth factor A, respectively (Figure 1G).

#### T Cell Markers

Expression of *CD2*, coding for CD2 molecule; *CD8A*, coding for T-Cell Surface Glycoprotein CD8 Alpha Chain; and *TRBC1*, coding for T-cell receptor beta constant 1, was significantly decreased in sALS cases. The expression of *CD44* and T-cell surface glycoprotein CD4 gene (*CD4*) was not modified (Figure 1H).

### Correlation between Clinical Parameters and Gene Transcription

**Figure 2:**
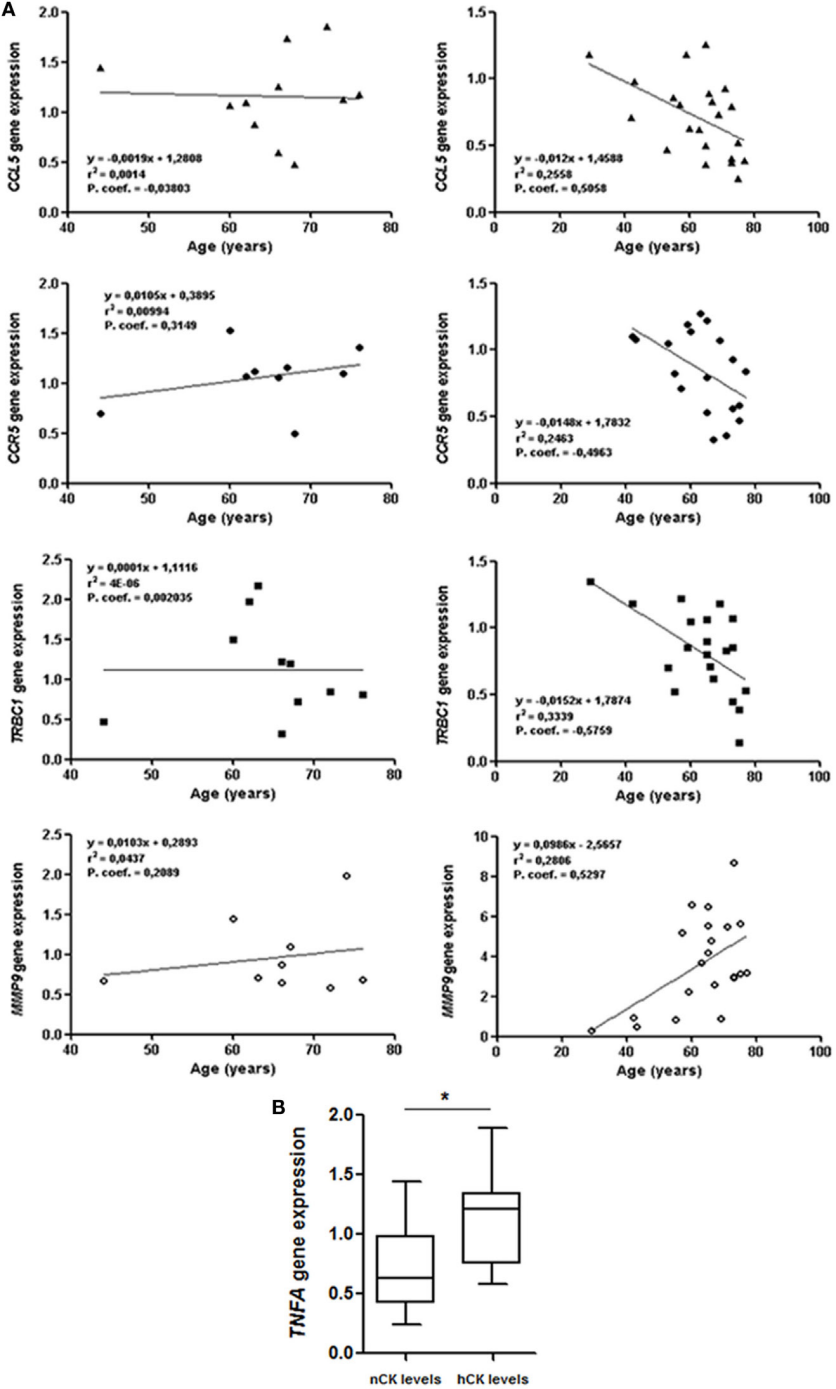
**(A)** Positive correlation between *MMP9* and age at sampling, and negative correlation between age at sampling and *CCL5, CCR5*, and *TRBC1* in sporadic amyotrophic lateral sclerosis (sALS) (right graphs), but not in control cases (left graphs). **(B)** Relation between *TNFA* mRNA expression levels in blood and creatine kinase (CK) protein levels in serum in sALS (nCK, normal CK levels; hCK, high/out of range CK levels) using Student’s *t*-test. Gene expression values correspond to fold change values of ΔΔCT.

Gender, ALS form of onset (spinal, bulbar, and respiratory), clinical progression, leukocyte counts and leukocyte types, and RIN values did not correlate with modifications in gene expression. However, *MMP9* levels in sALS cases positively correlated with age (*p* = 0.046) (Figure 2A). *CCR5* (*p* = 0.0307), *CCL5* (*p* = 0.016), and *TRBC1* (*p* = 0.0076) negatively correlated with age (Figure 2A) in sALS. These changes were not observed in the control group. Importantly, patients with sALS showed significant relation between elevated levels of *TNFA* gene and creatinine kinase (CK) values, which were out of the normal range (*p* = 0.025) (Figure 2B).

## Discussion

Peripheral inflammatory responses are common, but poorly defined, in human neurodegenerative diseases. Several studies focus on inflammatory responses in spinal cord and blood in sALS (1–11). The present study was geared to gain information about inflammatory gene expression profiles in the whole blood in a series of sALS patients at the beginning of clinical symptoms and non-treated with riluzole in order to avoid bias related to the treatment.

Present observations complement data from previous studies and point to the activation of mechanisms facilitating extravasation of WBC to target organs.

Neutrophil recruitment is supported by leukocyte adhesion molecules, chemokines, and cytokines (32, 33). Increased expression of *ITGB2* and a tendency of *ICAM1* to increase in blood suggest that adhesion and trans-endothelial migration of leukocytes is facilitated in sALS (34–36). Selectin 1, encoded by *SELL*, participates in leukocyte binding to endothelial cells and facilitates migration of WBC (37, 38); *SELL* expression has a tendency to increase in sALS. Increased expression of *MMP9* favors degradation of extracellular matrix components and facilitation of leukocyte migration (39). MMP9 is usually secreted in conjunction with TIMP-1, a specific inhibitor, which controls its proteolytic activity (40). A balance between MMP9 and TIMP-1 proteins regulates excessive tissue degradation in chronic inflammation (41). However, mRNA expression levels of cathepsins, also involved in extracellular matrix degradation (42), are not modified in blood of ALS cases when compared with blood samples from controls.

Expression levels of *CCL2* and *CCL3* v, the products of which modulate monocyte attraction (43, 44) are not modified in sALS. Moreover, reduced expression of *CCR5, CCL5*, and *CXCR5* supports reduced activation of B-cells (45).

The product of *CD2* expressed in T-cells modulates T-cell proliferation (46), whereas the product of *TRBC1* is implicated in T-cell activation (47). *CCL5* and *CCR5* encode T-cell chemo-attractant and regulatory molecules (48, 49). Reduced mRNA expression of these markers suggests inhibition of T-cell signaling.

Finally, increased *INPP5D* mRNA expression favors a negative regulation of myeloid cell proliferation (50).

Toll-like receptors are involved in the initiation of the inflammatory process (51). Reduced levels of *TLR3* accompanied by tendency to increased *TLR4* and *TLR2* mRNA expression point to ambiguous activation signaling by Toll-like receptors.

*TGFB2, IL10*, and *IL6* mRNAs are downregulated, and *IL10RA* and *TNFA* have tendency to decrease in blood in sALS when compared with controls. Expression levels of *IL10RB, TGFB1, IL1*β, *IL6ST, INFG* (coding for interferon γ), and *VEGFA* are not modified in sALS. Expression levels of assessed colony-stimulating receptors and *CSF3R* do not differ from control values. Even considering the increased expression of *TNFR1S* mRNA, the final scenario is downregulation of pro- and anti-inflammatory cytokines in sALS.

SOD1 transgenic mice lacking functional CD4+ T cells show increased motor neuron damage which is reversed following bone marrow transplants thus suggesting a neuroprotective role of CD4+ T cells (52). On the other hand, SOD1 transgenic mice with additional depletion of the Rag2 gene (mSOD1/RAG2−/− mice) show delayed motor neuron disease, thus suggesting that mature lymphocytes produce deleterious effects on vulnerable motor neurons (53).

Previous studies have shown a higher percentage of IL-13-positive CD4 and CD8 lymphocytes (8), increased numbers of peripheral CD8 cytotoxic T-cells and natural killer cells, together with decreased regulatory T (treg) lymphocytes (10) in ALS. Our observations show decreased expression of *CD2*, coding for CD2 molecule, *TRBC1*, coding for T-cell receptor beta constant 1 and *CD8* mRNA, and preserved *CD4* mRNA expression. Therefore, additional studies are necessary to elucidate these discrepancies in larger series.

The present findings show a complex scenario at early clinical stages of sALS, including on the one hand upregulation of genes whose products are involved in leukocyte extravasation and extracellular matrix remodeling, and on the other, downregulation of chemokines, anti- and pro-inflammatory cytokines, and lymphocyte modulators.

Positive correlation between *MMP9* and age, and negative correlation between age and *CCL5, CCR5*, and *TRBC1* has been observed in sALS but not in controls. No correlation has been found between present observations and first clinical manifestation, gender, and disease progression. Therefore, the present findings have little prognosis value.

There is only positive correlation between *TNFA* mRNA expression and CK levels. Although *TNFA* mRNA expression is lower in ALS when compared with controls, higher *TNFA* mRNA values correlate with higher CK protein levels. This observation points to the possibility of a link between *TNFA* and muscular damage in sALS. Previous studies have shown that muscular pathology is accompanied by increased expression of systemic inflammatory markers (17). Moreover, increased expression of inflammatory markers, including IL-1β and TNF-α, is found in the skeletal muscle at symptomatic and end-stages of SOD1(G93A) transgenic mice (18). However, these individual data are not sufficient to advance any definitive conclusion.

Transcriptome studies at early clinical stages in SOD1(G93A) transgenic mice have shown deregulated pathways common to spinal cord, muscle and sciatic nerve; two pathways are associated with T cell activation, two with macrophage activation, and one pathway contains genes involved in co-stimulatory regulation of the adaptive and innate immune systems; but blood did not show representation of these altered pathways (54). However, genetic ablation of IP3 receptor 2, which modulates inflammation and which expression is augmented in the spinal cord in ALS and related mice models, increases cytokines and decreases survival of SOD1G93A mice (55). These studies point to involvement of peripheral blood cells in the inflammatory response in the spinal cord in ALS. Present observations show systemic inflammatory responses linked to extravasation of leukocytes and remodeling of extracellular matrix at early stages of sALS. However, the observed changes do not indicate the primary or secondary origin, and the precise link between intrinsic and peripheral inflammatory responses in the pathogenesis of sALS.

## Ethics Statement

Blood samples from sALS cases and age-matched controls were obtained following signed informed consent and approval by Clinical Research Ethics Committee (CEIC) of the Bellvitge University Hospital.

## Author Contributions

All the authors designed, supervised the study, and wrote the final version of the manuscript.

## Conflict of Interest Statement

The authors declare that the research was conducted in the absence of any commercial or financial relationships that could be construed as a potential conflict of interest.
